# Thomas Paine

**Published:** 1892-11

**Authors:** 


### THOMAS PAINE.

Thomas Paine died on the 8th of July, 1809, at New Rochelle, N. Y., and was buried on an estate owned by him in that town, writes B. P., in the Investigator. In 1819, the noted Englishman, William Cobbett, exhumed his remains and took them to England. Soon after Cobbett's death in 1835, his effects were disposed of at public auction. Among them were the skull, bones and coffin-plate of Thomas Paine. The auctioneer, however, refused to sell them, saying it was out of his line to sell human bones, and they were withdrawn. In a sketch of Paine's life, in the last edition of Chamber's Encyclopedia, it is stated that these relics cannot be traced beyond 1847.

